# Microbiome bacterial influencers of host immunity and response to immunotherapy

**DOI:** 10.1016/j.xcrm.2024.101487

**Published:** 2024-03-27

**Authors:** Yeganeh Yousefi, Kelly J. Baines, Saman Maleki Vareki

**Affiliations:** 1 Verspeeten Family Cancer Centre, Lawson Health Research Institute, London, ON N6A 5W9, Canada; 2 Department of Pathology and Laboratory Medicine, Western University, London, ON N6A 3K7, Canada; 3 Department of Oncology, Western University, London, ON N6A 3K7, Canada

**Keywords:** gut microbiota, immunotherapy, immune checkpoint inhibitors, fecal microbiota transplantation, antibiotics, bacterial consortia, probiotics bacteria

## Abstract

The gut microbiota influences anti-tumor immunity and can induce or inhibit response to immune checkpoint inhibitors (ICIs). Therefore, microbiome features are being studied as predictive/prognostic biomarkers of patient response to ICIs, and microbiome-based interventions are attractive adjuvant treatments in combination with ICIs. Specific gut-resident bacteria can influence the effectiveness of immunotherapy; however, the mechanism of action on how these bacteria affect anti-tumor immunity and response to ICIs is not fully understood. Nevertheless, early bacterial-based therapeutic strategies have demonstrated that targeting the gut microbiome through various methods can enhance the effectiveness of ICIs, resulting in improved clinical responses in patients with a diverse range of cancers. Therefore, understanding the microbiota-driven mechanisms of response to immunotherapy can augment the success of these interventions, particularly in patients with treatment-refractory cancers.

## Graphical abstract

**Figure:**
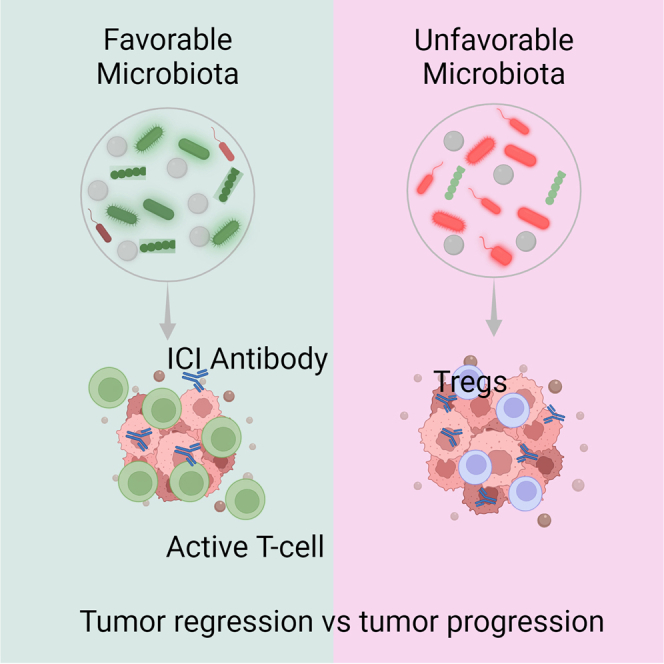

## Teaser

The gut microbiota shapes the immune system, which is important given the widespread use of immunotherapy drugs that work by activating the immune system. Yousefi et al. describe the bacteria commonly found in the gut, their role in shaping the immune response, and how it impacts response to immunotherapy.

## Introduction

Different human tissues, such as the gastrointestinal (GI) tract, lung, and skin, are habitats for numerous microorganisms (bacteria, viruses, fungi, protozoa, and archaea), with most organs having distinct microbial communities.^1^^,^^2^ The GI tract is predominantly populated by aerobic and anaerobic bacteria.^3^^,^^4^ While the composition and functionality of the microbiome varies among individuals, these microorganisms usually have a symbiotic relationship with their hosts that benefits both entities.^3^^,^^4^^,^^5^ For example, resident bacteria benefit from the protected and nutrient-rich environment in the gut.^6^ In return, the gut microbiota is engaged in various host physiological processes such as nutrient digestion and absorption,^7^^,^^8^ vitamin synthesis,^9^ and prevention of pathological colonization of the gut.^10^ More importantly, the host immune system relies on the gut microbiota for normal development and maintaining intestinal homeostasis.^4^^,^^11^ Therefore, gut microbiota dysbiosis, disrupting homeostatic microbiota-host interactions, is associated with various diseases, such as allergy,^12^ obesity,^13^^,^^14^^,^^15^ diabetes,^16^^,^^17^^,^^18^ inflammatory bowel disease,^19^ and cancer.^20^^,^^21^^,^^22^

The gut microbiota can impact carcinogenesis by disrupting signaling pathways involved in inflammation, DNA repair, and stability.^23^ Depending on the organ, bacterial-driven carcinogenesis is either caused by organ-specific microbiota or by effects of a distant bacterial community.^24^ For instance, *Helicobacter pylori*, which infects almost half of the world’s population, has a significant role in the onset of atrophic gastritis and the development of gastric cancer.^25^ On the other hand, several organs, such as the liver and pancreas, lack a recognized microbial community; therefore, exposure to bacterial components or metabolites can contribute to carcinogenesis in these organs.^23^ In contrast, bacteria can have anti-tumor effects through bacterial-derived ligands that bind to toll-like receptors (TLRs) and NOD-like receptors (NLRs) on various immune cells responsible for triggering innate immunity and, as a result, promoting anti-tumor immune responses.^23^^,^^26^^,^^27^ Immune mediators such as type I interferons (IFNs) are produced upon activation of TLRs and NLRs, redirecting tolerogenic immune responses toward anti-tumor immunity.^26^^,^^28^ TLRs, including TLR2 and TLR3, are being investigated in clinical trials as adjunctive therapies and primary treatment options.^29^ For example, it was found that a ligand associated with TLR1/TLR2 can inhibit T regulatory cells (Tregs), which in turn amplifies the activity of cytotoxic T lymphocytes.^30^ In addition, several research studies have verified the anti-cancer properties of TLR3 through its direct role in inducing apoptosis in malignant cells.^29^

Front-line cancer treatments are surgery, radiotherapy, chemotherapy, and immunotherapy.^31^ However, radiation and chemotherapy have limited specificity and may harm healthy tissues along with cancerous ones,^32^ whereas most immunotherapies activate T cells and eliminate cancer cells, leaving healthy bystander cells intact.^33^^,^^34^ Immune checkpoint inhibitors (ICIs) are among immunotherapy approaches that block immune inhibitory molecules on T cells.^35^ In particular, lymphocyte-associated antigen 4 (CLTA-4), programmed cell death protein 1 (PD-1), and programmed cell death 1 ligand 1 (PD-L1). These drugs have transformed cancer treatments by their capability to extend survival in patients with advanced cancers.^31^^,^^34^ However, treatment outcomes are variable, and not all patients experience therapeutic benefits.

Various tumor-dependent and -independent mechanisms affect the efficacy of ICI therapy. The gut microbiota is being exceedingly recognized as a contributing factor with considerable effects in regulating local and systemic immune responses in mouse models and human studies.^36^^,^^37^^,^^38^^,^^39^^,^^40^ For example, observational studies have exhibited an inverse correlation between antibiotic treatment and the positive outcomes of ICI administration,^41^^,^^42^^,^^43^^,^^44^ suggesting that gut microbiome integrity and gut-trained immunity regulate the effectiveness of ICI therapy. Studies have demonstrated that the abundance levels of specific bacteria in the gut microbiota greatly influence the host’s immune response and ICI efficacy.^8^^,^^43^^,^^45^ Microbial communities enriched in ICI responder patients and linked to improved efficacy of ICI treatments are regarded as "favorable" microbiota.^46^

On the other hand, bacterial composition and abundance in cancer patients who do not experience clinical response to ICIs are referred to as "unfavorable" microorganisms.^47^ Mice with favorable microbiota, including bacterial members of Ruminococcacea and Bifidobacteriaceae*,* compared with those with unfavorable microbiota, showed superior response after treatment with anti-PD-1/PD-L1 inhibitors.^43^^,^^48^^,^^49^ Routy et al. showed that higher diversity in the gut microbial communities is correlated to the success of anti-PD-1/PD-L1 treatment in patients with non-small cell lung carcinoma (NSCLC), renal cell carcinoma (RCC), or urothelial carcinoma.^43^ The presence of specific bacteria, such as *Faecalibacterium*, in patients' gut microbiota composition before receiving ipilimumab is associated with favorable clinical outcomes to treatment with this anti-CTLA-4 drug.^50^ Interestingly, according to preclinical^48^^,^^51^ and clinical^52^^,^^53^ evidence, the therapeutic advantages of beneficial bacteria may be transferable to patients by manipulating their microbial population, thereby rendering them responsive. Pioneering clinical trials have demonstrated that patients receiving fecal microbiota transplantation (FMT) and ICIs overcame resistance or experienced high clinical response to anti-PD-1 therapy in treating gastrointestinal cancers^54^ and melanoma.^46^^,^^52^^,^^53^ Since the gut microbiota is modifiable through diet, antibiotic treatment, probiotics, and FMT, these interventions may be utilized to improve ICI efficacy. This clinical opportunity has sparked great interest in studying host-microbiota interactions, their impacts on ICI therapy, and finding a plausible mechanism by which bacteria influence clinical response to ICI. Here, we describe how microbiota and specific bacteria in cancer patients may influence ICI efficacy by altering host immune responses and highlight the possibility of targeting these bacteria-driven immune responses to potentiate ICI treatment.

### Microbial crosstalk with the host immune system shapes ICI efficacy

Several human and mouse studies have demonstrated that gut microbiota impacts ICI effectiveness by modulating host immune responses.^43^^,^^49^^,^^55^ The influence of gut microbiota on ICI efficacy can be either immune-inhibiting or immune-activating. There are two main mechanisms for achieving immune-stimulatory effects: first, by reducing immune regulatory functions of Tregs and other immune suppressive cells, and second, by promoting local and peripheral T cell-mediated immunity toward tumors.^45^ For example, increased T cell recruitment to the tumors with higher activation and enhanced effector functions were attributed to the abundance of certain bacterial families such as Lachnospiraceae,^56^ Ruminococcaceae,^56^^,^^57^ Oscillospiraceae,^50^^,^^55^^,^^58^^,^^59^ and Akkermansiaceae.^43^ On the other hand, some bacteria, such as *H. pylori* downregulate anti-tumor immune responses by inducing immune evasion and inhibiting T cell activity.^60^

The microbiota-mediated immunosuppressive effects can be mediated by directly hindering anti-cancer immune activities via their cell wall components, i.e., lipoteichoic acid, and metabolites, i.e., short-chain fatty acids (SCFAs), or by intensifying the regulatory capacities of tumor-associated macrophages and Tregs.^61^ While Gao et al. discovered that intratumoral injection of *Fusobacterium nucleatum* improved the effectiveness of PD-L1 blockade in colorectal cancer, a contradictory study revealed that succinic acid derived from *F*. *nucleatum* reduced sensitivity to anti-PD-1 antibody in colorectal cancer by impairing CD8^+^ T cell-mediated immunity.^62^^,^^63^ Together, these two studies indicate that it is possible that the specific functions performed by the microbiota, rather than the presence or absence of a particular species, are more critical to the success of immunotherapy treatments. Moreover, a correlation between ICI response rates and the increased presence of intestinal microbial metabolites has been discovered.^64^ SCFAs are the principal source for metabolic activities of the gut microbiota and are produced by organisms such as Ruminococcaceae, *Lactobacillus,* and Bifidobacteriaceae via fermentation of indigestible food fiber and glycoproteins.^64^^,^^65^^,^^66^ Evidence shows that SCFAs such as acetic acid, propionate, butyrate, and valeric acid may improve clinical outcomes in response to ICI treatment.^67^^,^^68^ According to recent research, increased fecal SCFA concentration levels are linked to improved long-term response to anti-PD-1 treatment^69^ and more prolonged progression-free survival (PFS) upon receiving the same treatment.^67^ Therefore, it is essential to recognize that both bacterial species and bacterial metabolites can affect ICI outcomes. These microorganisms and their metabolites can function as potential biomarkers of response to ICI treatment or as factors with therapeutic effects on ICI therapy. Collectively, targeting the microbiota and their metabolites could be a successful strategy in reprogramming the tumor microenvironment and improving the effectiveness of ICI therapy.

### Microbiota affecting ICI results

#### Bacillota phylum (Firmicutes)

##### **Lachnospiraceae**

*Lachnoclostridium* bacteria belonging to the family Lachnospiraceae have anti-inflammatory properties as well as positive effects on liver cirrhosis and other liver disorders.^70^ However, its contributions to tumor suppression and ICI activity have not been thoroughly clarified. A recent study revealed that fecal samples of unresectable hepatocellular carcinoma (HCC) patients with objective clinical responses following treatment with anti-PD-1 agents (nivolumab or pembrolizumab) were enriched with *Lachnoclostridium* (Figure 2; Table 1).^71^ In responder patients, high concentration of certain bacterial metabolites, such as bile acids, including ursodeoxycholic acid and ursocholic acid, was also associated with the abundance of *Lachnoclostridium*.^71^ A similar enrichment of this bacterial population was also observed in melanoma patients who responded to anti-PD-1 therapy (Figure 2; Table 1).^72^

**Table 1:** Positive and negative impacts of different bacterial families on the efficacy of ICI treatments in preclinical and clinical studies

	Type of ICI treatment	Type of cancer	Influence on ICI treatment	Key immune cells	Observation	Reference
***Lachnoclostridium*** ***L. bacterium 3 1 46FAA***	Anti-PD-1	HCC, Melanoma	Positive	Enhanced CD8^+^ T cell infiltration	Improved PFS and prolonged overall survival	Peng et al.,^55^ Liu et al.,^62^ Hayase et al.,^65^ Temraz et al.,^76^ Hakozaki et al.^77^
***L. bacterium 5 1 57FAA***	Anti-PD-1	Melanoma	Negative	Unknown mechanism	Shorter PFS	Kim et al.^65^
**Ruminococcaceae** ***R. SGB15234*** ***R. SGB14909***	Anti-PD-1 Anti-CTLA-4	Melanoma, NSCLC, HCC, GC and CRC	Positive	Elevated levels of active CD4^+^ and CD8^+^ T cells	Better clinical response to ICI treatments, Higher PFS	Sivan et al.,^48^ Baruch et al.,^53^ Peng et al.,^55^ Peters et al.,^59^ Gong et al.,^60^ Cremonesi et al.,^79^ Wojas-Krawczyk et al.,^83^ Carbone et al.,^84^ Botticelli et al.^85^
***R. obeum*** ***R. gnavus*** ***R. bromii***	Anti-PD-1	Melanoma, Muscle-invasive urothelial carcinoma, NSCLC	Negative	Unknown mechanism	Non-responder to ICI treatments	Davar et al.,^52^ Kim et al.,^65^ Fitzgerald et al.^90^
					Shorter PFS	
					Less abundant in responder patients	
**Oscillospiraceae** ***F. prausnitzii*** ***F. SGB15346***	Anti-PD-1 Anti-CTLA-4 Combined therapy	Melanoma, NSCLC, and RCC	Positive	Facilitating the proliferation of CTLA4^+^ Tregs, Elevated intratumoral CD8^+^ T cells, peripheral CD8^+^ T cells, and effector CD4^+^ T cells	Enhance the effectiveness of ICI treatment	Baruch et al.,^53^ Peters et al.,^59^ Gong et al.,^60^ Hayase et al.,^64^ Kim et al.,^65^ Fitzgerald et al.,^90^ Frąk et al.,^95^ Romano et al.,^96^ Fu et al.,^97^ Dong et al.^101^
***F. prausnitzii***	Anti-PD-1	Melanoma	Negative	Unknown mechanism	Disease stabilization or progression	Katayama et al.^103^
**Bacteroidetes** ***B. ovatus*** ***B. dorei*** ***B. massiliensis***	Anti-PD-1 Anti-PDL-1 Anti-CTLA-4 Combined therapy	Melanoma, GI cancers, NSCLC, and HCC	Negative	Increased peripheral Tregs and MDSCs/Diminished peripheral cytokine responses	Shorter PFS, Lower response rates to ICI therapy	Baruch et al.,^53^ Gong et al.,^60^ Kim et al.,^65^ Fukuoka et al.,^78^ Lee et al.,^108^ Bender et al.,^110^ Elson et al.^111^
**Bacteroidetes** ***B. caccae*** ***B. fragilis*** ***B. Thetaiotaomicron*** ***B. salyersiae***	Anti-PD-1 Anti-CTLA-4 Combined therapy	Melanoma, and RCC	Positive	Enhanced the presence of T cell infiltrations within tumors and the frequencies of peripheral T cells	Extended PFS	Hayase et al.,^64^ Panda et al.,^112^ Louis et al.^113^
**Akkermansiaceae**	Anti-PD-1 Anti-PDL-1	Melanoma, NSCLC, HCC, and RCC	Positive	Triggering dendritic cells to release immune responses associated with Th-1 activation, resulting in the production of IL-12 and IFN-ɣ	Prolonged OS durations	Sivan et al.,^48^ Davar et al.,^52^ Cremonesi et al.,^79^ Li et al.,^118^ Derosa et al.^125^
	Anti-PD-1	mCRPC	Negative	Unknown mechanism	Depleted in responders	Derosa et al.^125^
**Coriobacteriaceae** ***C. aerofaciens***	Anti-PD-1	Melanoma	Positive	Unknown mechanism	More abundant in responder patients	Davar et al.^52^
**Bifidobacteriaceae** ***B. adolescentis*** ***B. Longum*** ***B. Bifidum***	Anti-PD-1	Melanoma, NSCLC, CRC and RCC	Positive	Activation of CD8^+^ T cells, Reduced Tregs activation	Diminished tumor advancement	Davar et al.,^52^ Oliva et al.,^87^ Gao et al.,^91^ Haikala et al.,^131^ Han et al.^133^
**Lactobacillaceae** ***L. reuteri***	Anti-PD-1 Anti-PDL-1	Melanoma, GI cancers, HCC, NSCLC	Positive	Improved infiltration of CD8^+^ T Cells in mice fed with Lactobacillaceae	Elevated therapeutic outcomes of ICI treatments	Davar et al.,^52^ Gong et al.,^60^ Fukuoka et al.,^79^ Zhao et al.,^109^ Kawahara et al.,^143^ Rizvi et al.,^145^ Gihawi et al.^148^

In a different human cohort involving melanoma patients, it was observed that the presence of the Lachnospiraceae bacterium strain *3 1 46FAA* in fecal samples of patients receiving anti-PD-1 treatment was linked to improved PFS (Figure 2; Table 1).^59^ On the other hand, the group with a higher concentration of Lachnospiraceae bacterium strain *5 1 57FAA* experienced an increased risk of disease progression (Figure 2; Table 1).^59^ Furthermore, Lachnospiraceae were detected in both donors and recipient responder patients^52^ of the two clinical trials that demonstrated that combining FMT from ICI responders with anti-PD-1 therapy can overcome resistance to PD-1 blockade in refractory melanoma patients.

To further understand how Lachnospiraceae can elicit its effects, Zhu et al. showed that enhanced CD8^+^ T cell infiltration may account for the correlation between the amount of intertumoral *Lachnoclostridium* genus and prolonged overall survival (OS) in patients with advanced cutaneous melanoma (Table 1).^56^ These clinical studies emphasize the positive impact of the abundance of Lachnospiraceae in fecal samples of patients receiving ICI, in particular anti-PD-1 treatment, on improved outcomes. The mechanisms through which Lachnospiraceae impact the effectiveness of ICI treatments are still unclear. Further investigation is needed into how this family of bacteria interacts with the immune system and ICI therapies. Moreover, establishing specific biomarkers that indicate a patient’s reaction to ICI therapy through the baseline levels of Lachnospiraceae could assist in customizing treatment plans for individual patients.

##### **Ruminococcaceae**

**Figure 1:**
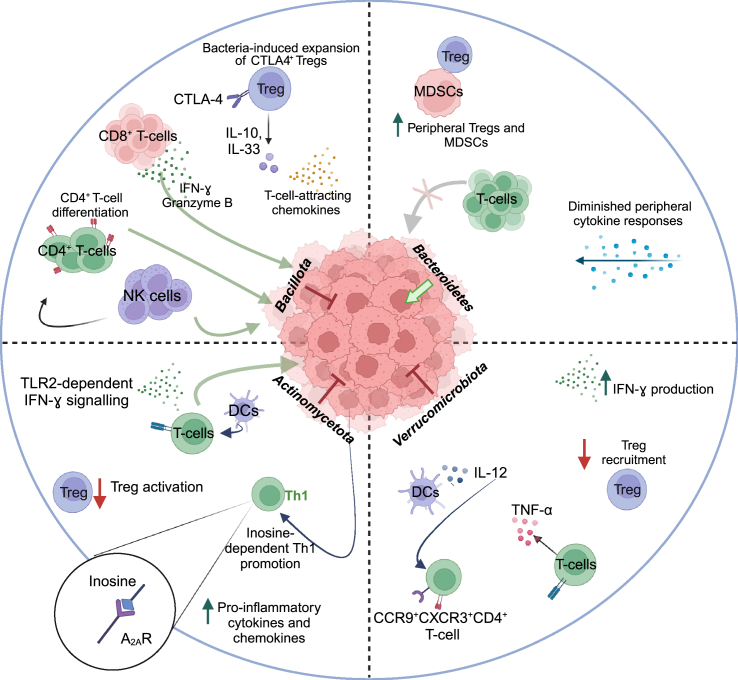
Microbiota-associated mechanistic pathways underlying either anti-tumor efficacy or tumor progression effects (A) A correlation exists between the presence of specific Bacillota family members and elevated levels of effector CD4^+^ and CD8^+^ T cells, the secretion of cytokines like IL-10 and IL-33, and the generation of T cell-attracting chemokines. (B) Bacteroidales is inversely correlated with elevated T cell infiltration in tumors and peripheral T cell counts, while also dampening peripheral cytokine reactions and boosting frequencies of peripheral Tregs and MDSCs. (C) Clinical outcomes after FMT with *A. muciniphila* are linked to Th-1-related immune responses. (D) The combined administration of *Bifidobacterium* and anti-PD-1 mitigates tumor growth by targeting T-cell-specific A_2A_R pathways, enhancing CD8^+^ T cell activation, and diminishing Tregs activation. (E) Lactobacillaceae stimulates immune reactions linked to enhanced anti-tumor effects. This includes increased production of IFN-γ and Granzyme B, heightened infiltration of CD8^+^ T cells and NK cells into tumor sites, and encouragement of Th-1-type CD4^+^ differentiation. Green arrows represent an increase in the mentioned cell population, while red arrows indicate a decrease. Tregs, regulatory T cells; MDSCs, myeloid-derived suppressor cells; IFN-ɣ, interferon-gamma; A_2A_R, adenosine 2A receptor; TLR2, Toll-like receptor 2.

**Figure 2:**
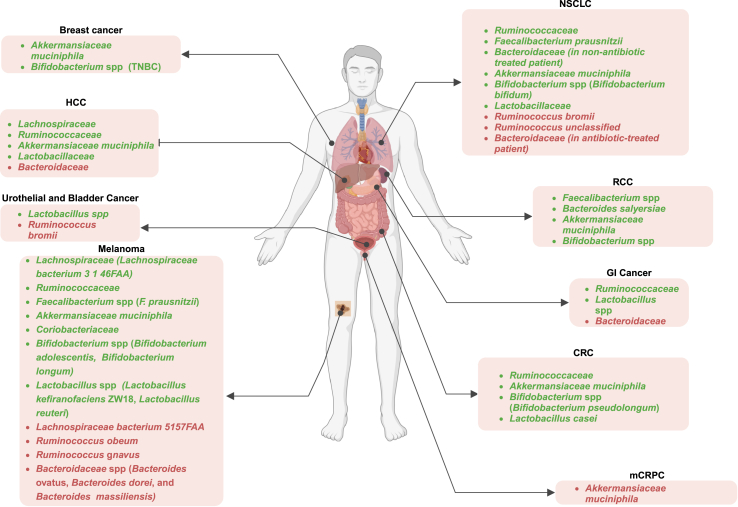
Association between bacterial population and the outcomes of ICI treatments The presence or high abundance of green-highlighted bacteria among cancer patients correlates with positive responses to ICI treatments across different cancer types, whereas the prevalence of red-highlighted bacteria is higher in non-responder patients. TNBC, triple-negative breast cancer; HCC, hepatocellular carcinoma; NSCLC, non-small cell lung carcinoma; RCC, renal cell carcinoma; GI, gastrointestinal; CRC, colorectal cancer; mCRPC, metastatic castrate-resistant prostate cancer.

Commensal Ruminococcaceae promote host health by decreasing intestinal permeability.^73^ The Ruminococcaceae family also plays a significant role in generating SCFAs such as acetate and propionate.^74^ Two independent studies using 16s rRNA gene sequencing and metagenomics shotgun sequencing on fecal samples of NSCLC patients showed that *Ruminococcus* spp were enriched in patients with better clinical responses than those with shorter PFS.^43^^,^^75^ The beneficial influence of this bacterial family in ICI responsiveness has been reported in several cancer types, including melanoma,^46^^,^^52^^,^^55^ NSCLC,^43^^,^^75^ HCC,^73^ and colorectal cancer (Figure 2; Table 1).^76^ In a study by Gopalakrishnan et al., enrichment of Ruminococcaceae in melanoma patients was correlated with increased levels of effector CD4^+^ and CD8^+^ T cells and responsiveness to anti-PD-1 (Figure 1; Table 1).^55^ Similarly, it has been shown that the presence of *Ruminococcus* in melanoma patients was predictive of response to treatments with both ipilimumab (anti-CTLA-4) and pembrolizumab (Table 1).^50^^,^^52^ Melanoma patients with baseline *Bacillota*-dominant microbiota (including *Ruminococcus* and Lachnospiraceae) experienced an improved clinical response to ipilimumab and longer OS (Table 1).^50^ Results from our recent phase I clinical trial were consistent with these findings, showing an increased presence of Ruminococcaceae SGB15234 and SGB14909 in melanoma patients who responded to treatment with healthy donor FMT, plus nivolumab or pembrolizumab treatment (Table 1).^46^ The predictive advantage was also demonstrated in two independent studies that analyzed fecal samples of NSCLC (n = 70), NSCLC (n = 14), and gastric cancer (n = 24) patients, confirming that patients with Ruminococcaceae enrichment had a robust clinical response to PD-1 blockade (Figure 2; Table 1).^77^^,^^78^ In another study on HCC patients, *Ruminococcus* spp were enriched in responder patients upon camrelizumab (anti-PD-1) treatment (Figure 2; Table 1).^73^ In addition to human clinical studies, xenograft examination of human colorectal cancer tumors revealed a correlation between the presence of certain *Bacillota* family members, particularly Lachnospiraceae and Ruminococcaceae, and the production of T cell-attracting chemokines, including chemokine (C-C motif) ligand 5 (CCL5) and 20, CCL20 and CXC Motif Chemokine Ligand (CXCL) 11 (Figure 1; Table 1).^79^ However, the expression of these chemokines in tumor-bearing mice was notably decreased after antibiotic treatment,^79^ suggesting that the primary inducers of chemokines in colorectal cancer are commensal bacteria.

Despite Ruminococcaceae improving ICI efficacy in multiple studies,^43^^,^^52^^,^^55^^,^^73^^,^^76^ Ruminococcaceae has been found to be implicated in increased ICI toxicity.^50^^,^^80^ Despite the established correlation between SCFAs and ICI responsiveness,^67^^,^^69^ and the fact that Ruminococcaceae is a significant producer of SCFAs, there is limited evidence suggesting that the beneficial impact of this bacterium on ICI outcomes is directly related to SCFAs.^81^

On the other hand, conflicting findings indicate that the increased abundance of some *Ruminococcus* spp*,* such as *Ruminococcus obeum* is linked to a lack of response to anti-PD-1 immunotherapy in metastatic melanoma (Figure 2; Table 1).^49^ Another melanoma study showed that increased *Ruminococcus gnavus* levels were correlated with a shorter PFS (Figure 2; Table 1).^59^ *Ruminococcus bromii* and *Ruminococcus unclassified* were also observed to be less prevalent in NSCLC patients responding to anti-PD-1 therapy (Figure 2; Table 1).^82^^,^^83^^,^^84^^,^^85^ Similarly, Pederzoli et al. reported that patients with muscle-invasive urothelial carcinoma who were unresponsive to anti-PD-1 had a higher prevalence of *R. bromii* (Figure 2).^86^ Therefore, identifying and characterizing the varying effects of Ruminococcaceae species on ICI therapy could support the development of biomarkers to predict treatment response based on the abundance and composition of these bacteria in the gut microbiome of cancer patients.

Collectively, research to date has shown that Ruminococcaceae is greater in patients who respond to anti-PD-1 or anti-CTLA-4 treatments across a variety of cancer types and may have potential as a therapeutic adjuvant to ICI treatments. In a recent human trial (NCT03817125), 14 patients with metastatic melanoma were treated with vancomycin followed by a combination of Ruminococcaceae-enriched bacterial communities and anti-PD-1 in the first line setting.^87^ Notably, patients in the combination treatment arm experienced a lower objective response rate (25%) than those in the anti-PD-1-only arm (66.7%). These findings imply that previous antibiotic treatments, particularly in the context of limited consortia therapy, might be harmful when patients receive bacterial-based interventions.^87^ Therefore, additional research is essential to identify the most effective and safe approach to adjusting the microbiota without risking patients' well-being.

##### **Oscillospiraceae**

Members of the Oscillospiraceae family are often recognized as part of healthy gut microbiota in humans.^88^ Oscillospiraceae includes the genus *Faecalibacterium* that comprises three species: *F. longum*, *F. butyricigenerans*, and *F. prausnitzii*.^89^ Studies employing 16S rRNA gene sequencing and metagenomics analysis identified *F. prausnitzii* as highly prevalent within the human gut, representing 5%–15% of the entire bacterial population.^90^ Several observational studies have concluded that the abundance of Oscillospiraceae*,* especially *F. prausnitzii,* positively correlates with ICI response in melanoma patients and decreases in adverse events (Figure 2; Table 1).^50^^,^^91^ For example, baseline enrichment of *Faecalibacterium* spp was linked with a positive response to anti-CTLA-4 in metastatic melanoma (Table 1).^50^^,^^68^ Other studies in melanoma patients have shown that patients with higher levels of *Faecalibacterium* spp (in particular *F. prausnitzii*) during treatment with anti-PD-1, anti-CTLA-4, or a combination of both experienced better clinical outcomes (Figure 2; Table 1).^46^^,^^55^^,^^58^^,^^59^^,^^92^ Additionally, NSCLC and RCC patients who responded positively to anti-PD-1 had a higher level of *Faecalibacterium* spp in their fecal samples compared with non-responders (Figure 2; Table 1).^85^

*Faecalibacterium* has been found to stimulate the proliferation of Tregs and release of some cytokines such as interleukin (IL)-10 and IL-33 (Figure 1), which help maintain a balance of anti-inflammatory factors in the intestines.^93^^,^^94^^,^^95^ Beneficial effects of *Faecalibacterium* are associated with an initial lower proportion of CD4^+^ Tregs, which is also linked to enhanced activity of anti-CTLA4 in patients.^50^ Interestingly, an *ex vivo* examination of peripheral blood mononuclear cells obtained from patients with advanced cutaneous melanoma showed that Tregs had high levels of CTLA-4, potentially rendering them more susceptible to depletion by ipilimumab (Table 1).^96^ These results suggest that *Faecalibacterium* can potentially augment the effectiveness of anti-CTLA-4 treatment by inducing the expansion of CTLA-4^+^ Tregs and could be used as an adjuvant to boost the efficacy of anti-CTLA-4 therapy. Besides, patients with higher levels of *Faecalibacterium* demonstrated an increase in the expression of inducible T cell co-stimulator (ICOS) on T cells, which has been reported as a potential biomarker for the effectiveness of ipilimumab treatment in cancer patients.^50^^,^^97^

In addition to anti-CTLA-4, it has been reported that *Faecalibaccterium* can enhance the efficacy of anti-PD-1 treatment. Gopalakrishnan et al. showed that the *Faecalibacterium*-derived effects were linked to increased frequencies of immune cells (such as intratumoral CD8^+^ T cells, peripheral CD8^+^ T cells, and effector CD4^+^ T cells (Figure 1; Table 1), along with higher cytokine concentrations, which enhance the efficacy of anti-PD-1 treatment.^55^ However, one study reported increased presence of *F. prausnitzii* at baseline can either stabilize or worsen the disease in patients with stage III or stage IV cutaneous melanoma who underwent anti-PD-1 treatment (Figure 2; Table 1).^98^ The variation in findings among studies about key species implicated in immunotherapy response may be attributed to limited sample size, methods used for microbiota analysis, and variances in the populations studied.

##### **Lactobacillaceae**

Commensal *Lactobacillus* members exhibit immunomodulatory characteristics and are frequently used as probiotic strains.^99^ Preclinical studies in melanoma, intestinal, and bladder cancers have reported that mice monocolonized with *L. johnsonii* were more responsive to anti-CTLA-4 (Figure 2).^100^ Diosgenin, a plant-based steroidal saponin, sensitized B16 melanoma tumor-bearing mice to anti-PD-1 by enriching the population of *L. genus* (Figure 2).^101^ Two clinical studies in melanoma patients reported a greater abundance of *Lactobacillus* spp in anti-PD-1/PD-L1 responders (Table 1).^49^^,^^55^ Peng and colleagues also showed that advanced-stage GI cancer patients who responded to anti-PD-1/PD-L1 treatments had a greater abundance of *Lactobacillus* in their fecal samples compared with those who did not respond to the same treatments (Figure 2).^102^ Similarly, HCC and NSCLC patients responsive to anti-PD-1 had a higher abundance of *Lactobacillus* spp in their gut microbiome compared with non-responders (Figure 2; Table 1).^73^^,^^103^ As there is no evidence suggesting that higher levels of *Lactobacillus* spp are linked to lowered effectiveness of ICIs, this bacterium could potentially serve as a biomarker for predicting the success of ICI treatments, highlighting the need for more clinical studies to prospectively establish the role of *Lactobacillus* spp in ICI treatment efficacy.

Several studies have investigated the immunomodulatory roles of *Lactobacillus* spp, which can shape anti-tumor immunity and ultimately enhance the effectiveness of ICI therapies. Co-culture of live *Lactobacillus* strains and species with mouse dendritic cells (DCs) promoted maturation of these cells through the induction of pro-inflammatory cytokines and expression of co-stimulatory molecules.^104^ Mice fed with *L. casei* supplementation showed an enhanced immune response and upregulated IFN-γ and Granzyme B production, as well as increased CD8^+^ T cell infiltration of tumors, which was associated with decreased tumor growth compared with controls (Figure 1; Table 1).^105^ In a similar mouse study, the administration of *L. plantarum* increased CD8^+^ cell infiltration and IFN-γ production, and natural killer (NK) cell infiltration into tumor tissues and promoted the differentiation of Th-1-type CD4^+^ T cells (Figure 1).^106^ Recently, researchers administered *L. rhamnosus GG* (LGG) to mice and observed increased activation of CD8^+^ T cells relies on the involvement of DCs and, more precisely, requires the expression of TLR2 on the surface of these cells.^107^ This TLR2-dependent mechanism for inducing immunomodulatory effects has also been observed in certain strains of *Bifidobacterium*.^108^ Unlike previous research,^105^^,^^106^ the administration of LGG was used as a therapeutic approach after the tumors had already been established to produce more clinically relevant results. A recent preclinical study revealed that oral administration of *L. kefiranofaciens* ZW18 (ZW18) effectively enhances the impact of anti-PD-1 therapy against melanoma.

Interestingly, the supplementation of ZW18 to mice treated with PD-1 inhibitors resulted in an optimized gut microbiota composition, with a significant rise in the levels of *Akkermansia*, Prevotellaceae_NK3B31 group, and *Muribaculum*.^109^ Similarly, ZW18 can potentially improve the effectiveness of PD-1 inhibitors for treating melanoma by increasing the infiltration of CD8^+^ T cells and boosting IFN-γ expression in tumor tissues.^109^ Besides, Bender et al. discovered that oral administration of *L. reuteri* can inhibit the growth of melanoma tumors in mice and enhance the efficacy of treatment with anti-PD-1 and anti-CTLA-4.^110^ This bacterium, *L. reuteri,* colonizes tumor tissue and produces indole-3-aldehyde (I3A), which activates a specific signaling pathway, aryl hydrocarbon receptor (AhR), in CD8^+^ T cells. This activation leads to an increase in the production of IFN-γ and Granzyme B.^111^ This study proposes a hypothesis that the gut microbiota, beyond its known indirect effects on immune responses and effectiveness of immunotherapy, can directly impact tumor immunity and responses to ICI therapies by translocating into the tumor microenvironment. In addition, the activation of the AhR by different molecular compounds can lead to contrasting consequences, including both tumor-suppressing and tumor-promoting.^111^ These observations indicate that the involvement of AhR activation in tumor immunity is dependent on the particular ligands.^110^^,^^111^ Therefore, these results emphasize the significance of investigating the communication between bacterial ligands and AhR in tumor tissues. Elucidating effects of microbiota-derived ligands on AhR activation within CD8^+^ T cells will not only improve our understanding of the complex relationship between the gut microbiota and the immune system, but it may also help identify novel therapeutic targets for enhancing anti-tumor immunity and improving cancer treatment outcomes. Overall, probiotics show potential as a therapeutic intervention in numerous preclinical studies, yet further investigation is crucial to pinpoint their exact clinical applications and confirm their effectiveness and safety.

#### Bacteroidota phylum (Bacteroidetes)

##### **Bacteroidetes**

The Bacteroidetes phylum contains gram-negative and anaerobes bacteria, most of which are from the *Bacteroides* genus in the human gut.^112^ Bacteroidota is the second most abundant bacterial phylum in the human gut, following Bacillota.^113^ Several studies have reported that systemic antibiotic treatment can disrupt this proportion and lead to a rise in the Bactceroidetes over the Bacillota.^41^^,^^112^^,^^114^ Such an imbalance could result in dysbiosis, negatively impacting the microbiome health and anti-tumor immunity. It has been reported that *Bacteroides* spp have extensive interactions with the host immune system, indicating they may influence immune responses and homeostasis of the host.^115^ Several studies have investigated the correlation between the abundance of Bacteroidetes and immune responses to ICI treatments. Liang et al. found that melanoma patients with higher levels of Bacteroidetes displayed a lower response rate to immunotherapy treatments.^116^ Patients with GI cancers who responded positively to anti-PD-1/PD-L1 treatment had lower *Bacteroides* genus levels than non-responder patients (Figure 2).^102^ Consistent with previous findings,^41^^,^^112^^,^^114^ patients diagnosed with stage IIIB or IV NSCLC had an increased abundance of Bacteroidota following antibiotic use, and patients who were untreated with antibiotics had a longer PFS of 16.7 months, compared to that of the entire group (14.3 months), regardless of whether they received antibiotics or not (Figure 2; Table 1).^117^ Increased Bacteroidales in the gut microbiota of non-responder patients experiencing shorter PFS were also noted in a cohort of HCC patients treated with anti-PD-1 antibody (Figure 2; Table 1).^118^ Consistent with previous results, multiple distinct cohorts of melanoma patients who received anti-PD-1, anti-CTLA-4, or combined therapy showed that Bacteroidaceae species have a negative correlation with responsiveness. Their findings demonstrated that *B. ovatus*, *B. dorei*, and *B. massiliensis* were more prevalent in non-responders with shorter PFS (Figure 2; Table 1).^50^^,^^55^^,^^59^^,^^72^

In contrast, a couple of clinical cohorts found that *B. caccae*, *B. fragilis*, and/or *B. thetaiotaomicron* were able to induce immune-stimulating effects in metastatic melanoma patients undergoing either monotherapy or combination therapy (Table 1).^58^^,^^119^ In addition, *B. salyersiae* showed higher abundance in anti-PD-1 responding RCC patients (Figure 2; Table 1).^120^ According to several clinical studies, the success rate of ICIs in several cancer types, including GI cancers,^102^ NSCLC,^117^ HCC,^118^ and melanoma,^50^^,^^55^^,^^59^ was shown to be negatively correlated with the enrichment of Bacteroidota. Bacteroidales attenuated peripheral cytokine responses and promoted frequencies of peripheral immunosuppressive immune cells, including Tregs and myeloid-derived suppressor cells (Figure 1; Table 1).^55^

Although many clinical studies have demonstrated a negative association between the abundance of Bacteroidetes and clinical outcomes, it has been reported that certain Bacteroidetes species have favorable effects on the host immune system. For instance, *B. fragilis* can exert beneficial effects, including increased CD4^+^ T cells on the host immune system, which may improve anti-tumor immunity.^121^^,^^122^ The advantageous impacts of Bacteroidetes species on the immune system, along with their prevalence and consistency in colons, make them highly suitable choices for use in a bacterial consortium. While the abundance of Bacteroidetes species has been linked to poor response rates, some research studies have indicated that incorporating specific *Bacteroides* spp into a bacterial consortium can enhance the effectiveness of ICI treatments by promoting effector immune responses. In a preclinical model of colorectal cancer, the addition *Bacteroides* spp into a bacterial consortium increased the frequency of IFN-γ^+^CD8^+^ tumor-infiltrating lymphocytes, which positively influenced the efficacy of anti-PD-1.^123^ However, currently, there is a lack of similar data in human studies. More studies are needed to investigate whether incorporating *Bacteroides* spp in a bacterial consortium can enhance the anti-tumor immune response and ultimately increase the efficacy of ICI therapies in clinical cancer patients.

#### Verrucomicrobiota

##### **Akkermansiaceae**

*Akkermansia muciniphila* is a bacterium that thrives in the digestive systems of both humans and animals. It is classified as a gram-negative, strictly anaerobic microorganism, and its primary function is to break down mucins.^124^ *A. muciniphila* was detected in greater abundance in the fecal microbiota of patients with melanoma,^49^ HCC,^73^ NSCLC,^43^^,^^125^ and RCC^43^ who responded to anti-PD-1/PD-L1 treatment (Figure 2; Table 1). Both antibiotic-treated mice and those treated with FMT from non-responder patients regained their responsiveness to anti-PD-1 therapy following oral administration of *A. muciniphila*.^43^ T helper-1 (Th-1)-related immunity, such as increased IFN-ɣ production, was the only immune response that was associated with PFS (Figure 1; Table 1). *A. muciniphila* has been linked to augmenting immune responses, by stimulating DCs to release IL-12 as well as decreasing the recruitment of immunosuppressive Tregs into the tumor microenvironment (Figure 1; Table 1).^43^ This cytokine is required to recruit CCR9^+^CXCR3^+^CD4^+^ T cells to the tumor microenvironment, thus increasing anti-PD-1 effectiveness (Figure 1).^43^ Findings from a large cohort of NSCLC patients treated with anti-PD-1 confirmed that the presence of *A. muciniphila* is related to greater objective response rates and longer OS (Figure 2; Table 1).^48^ *A. muciniphila* can also stimulate the recruitment of T cells and IFN-ɣ gene expression in the tumor microenvironment.^125^

Furthermore, *A. muciniphila* can modulate TLR1-TLR2 molecular pathways by producing a lipid in their cell membrane, diacyl phosphatidylethanolamine, which is recognized by TLR1-TLR2 heterodimers.^126^ It has also been well-documented that *A. muciniphila* plays a crucial role in host metabolic regulation.^127^ In a preclinical model of mouse microsatellite stable (MSS) colorectal cancer, microbiota composition altered by various antibiotic regimes was shown to respond differently to anti-PD-1 therapy, and the *A. muciniphila*-enriched antibiotic-treated group had a better response to anti-PD-1 by influencing the metabolism of glycerolipid (Figure 2).^128^ Moreover, in addition to activating cytotoxic T lymphocytes in the mesenteric lymph nodes, *A. muciniphila* can remodel the tumor microenvironment in a mouse model of colorectal cancer, thereby enhancing the immune response.^129^ Treatment with metformin, an anti-diabetes medication, has been shown to alter the gut microbiota composition and increase the abundance of *A. muciniphila*,^130^ improving the efficacy of anti-PD-1 in a model of MYC-driven breast cancer (Figure 2).^131^ Despite this, a recent study revealed that an *A. muciniphila*-dominated gut microbiota may be an indicator of subpar responses to anti-PD-1 blockade since NSCLC patients with a lower relative abundance of fecal *A. muciniphila* (between 0.035% and 4.799%) exhibited longer OS compared with those with a high relative abundance of *A. muciniphila.*^126^ Similarly, 16S rRNA gene sequencing and qPCR results from patients with metastatic castrate-resistant prostate cancer showed that *A. muciniphila* levels were lower in anti-PD-1 responders (Figure 2; Table 1).^132^

Considering that multiple studies^43^^,^^49^^,^^73^^,^^125^ have validated the positive effects of *A. muciniphila* on clinical outcomes after ICI treatments, it is plausible to consider this bacterium as a predictive biomarker. Oral administration of *A. muciniphila* has been shown to convert non-responders into responders in preclinical models^43^; therefore, incorporating *A. muciniphila* into a bacterial consortium could be a viable strategy to enhance the effectiveness of ICIs. Nonetheless, the dosage of this treatment plays a critical role since lower concentrations of *A. muciniphila* in the gut microbiota have demonstrated superior responses compared to gut microbiota dominated by *A. muciniphila*.^125^

#### Actinomycetota

##### **Coriobacteriaceae**

The *Collinsella* genus, which is part of the Coriobacteriaceae family, is identified as gram-positive and anaerobic.^133^ The potential advantage of *C. aerofaciens* in ICI treatment has only recently been investigated. Matson et al.^49^ found *C. aerofaciens* to be more abundant in melanoma patients who responded to anti-PD-1 treatment and that reconstituted germ-free mice with responder microbiota, including *C. aerofaciens*, had enhanced T cell activation compared with animals that received FMT from non-responders, which resulted in enhanced anti-PD-1 activity and decreased tumor growth (Figure 2; Table 1).^49^

In addition to boosting T cell activation, *C. aerofaciens* expansion promotes pro-inflammatory environments by raising IL-17A and CXCL1 and CXCL5 chemokines production.^134^ Additionally, in the CACO-2 colorectal cancer cell line, *C. aerofaciens* reduced expression of the tight junction proteins, including ZO-1 and occluding, leading to increased gut permeability.^134^ The integrity of the intestinal barrier is crucial, and its impairment is linked to the development of cancer.^135^ Therefore, due to the various immunomodulatory effects of *C. aerofaciens*, further explorations are required to identify the significance of this bacterium on ICI activity.

##### **Bifidobacteriaceae**

*Bifidobacteria* are gram-positive microorganisms that inhabit the human GI tract as part of its natural bacterial community.^136^ *Bifidobacterium* and *Lactobacillus* are the predominant microorganisms utilized as commercial probiotics.^137^ Findings from several studies demonstrated the presence of Bifidobacteriaceae family members is linked to enhanced immune-mediated tumor suppression and efficacy of ICI treatments (Table 1).^48^^,^^49^^,^^100^ The disparity in the effectiveness of anti-PD-L1 antibody in treating melanoma tumors in mice from two separate animal facilities was credited to the various levels of *Bifidobacterium* presence in those animals (Figure 2). This bacterium was found in notably higher proportions in JAX mice compared with TAC mice, and it was linked to enhanced anti-tumor T cell responses.^48^

*Bifidobacterium* is linked to enhanced effectiveness of ICI treatment in various types of cancers, including RCC,^86^ colorectal cancer,^100^ metastatic melanoma,^48^^,^^49^ NSCLC,^82^^,^^108^ and triple-negative breast cancer (TNBC) (Figure 2; Table 1).^138^ Treatment of colorectal cancer mouse models with anti-PD-1 or anti-CTLA-4 revealed that monocolonization with *B. pseudolongum* was sufficient to improve the effectiveness of ICI treatments (Figure 2).^100^ A clinical trial on EDP150, a *Bifidobacterium* strain, showed that the oral intake of this strain, combined with pembrolizumab, among patients with metastatic MSS colorectal cancer, was safe and well-tolerated. Mechanistically, this combination treatment reduced tumor progression by boosting the activation of CD8^+^ T cells and reducing Tregs activation within the tumor microenvironment (Figure 1; Table 1).^139^ A preclinical study on melanoma-bearing mice demonstrated that oral administration of *Bifidobacterium* as a single treatment enhanced tumor control to a level comparable to that achieved with PD-L1 alone. The combined approach bolstered the effectiveness of PD-L1 treatment and almost eliminated tumor expansion.^48^

Additionally, *B. adolescentis* and *B. longum* were enriched in melanoma patients with better clinical outcomes following anti-PD-1 treatment (Figure 2; Table 1).^49^ Interestingly, colonization of germ-free mice with responder fecal microbiota, including *B. longum* validated these clinical results, showing that responder microbiota reduced tumor growth and enhanced anti-PD-1 activity.^49^ An in-depth analysis of intestinal microbiome samples from individuals diagnosed with NSCLC revealed that those who experienced positive outcomes following anti-PD-1 treatment had a considerable abundance of *B. bifidum* (Figure 2; Table 1).^108^ Another preclinical investigation using the 4T1 TNBC model suggested that combining *B. longum RAPO* with anti-PD-1 potentially improves anti-cancer immune responses.^138^ In the group that received a combination treatment of *B. longum RAPO* with anti-PD-1, researchers observed higher CD8/CD4 T cell ratio levels in the spleen and increased NK cell levels within the tumor. In addition, the group that received combined treatments showed a decrease in pro-tumor-associated macrophages and an increase in anti-tumor cytokines (IFN-γ and TNF-α).^138^

The mechanisms through which *Bifidobacterium* plays a role in boosting anti-tumor responses have been the subject of many investigations. Sivan et al. discovered oral administration of *Bifidobacterium* in mice with B16·SIY melanoma tumors could modify the functions of DCs, consequently promoting the infiltration of CD8^+^ T cells into tumor tissues and improving tumor control to a level comparable to that achieved with anti-PD-L1 therapy.^48^ This is consistent with prior studies' results linking *Bifidobacterium* spp with increased IFN-ɣ production.^140^^,^^141^^,^^142^^,^^143^ Furthermore, Lee et al. discovered that the cooperative effects between specific strains of *Bifidobacterium* and anti-PD-1 inhibitors in reducing tumor size in mice rely on IFN-γ signaling, which is dependent on TLR2 (Figure 1). This study found that the critical factor determining the strain-specific synergistic impact of *Bifidobacterium* on cancer treatments is the peptidoglycan-mediated IFN-γ signaling pathway.^108^ Additionally, *Bifidobacterium* can also hinder tumor growth through antigen cross-reactivity. The epitope SVYRYYGL (SVY) is expressed by *B. breve* and is cross-reactive with a model neoantigen, SIYRYYGL (SIY), rendering tumors expressing SIY more susceptible to T-cell-mediated destruction. As a result, mice lacking *B. breve* experience increased tumor growth.^144^

A comprehensive metabolic analysis by Mager et al. revealed that inosine, a metabolite produced by *Bifidobacterium*, enhanced anti-tumor capacities and amplified the effects of anti-CTLA-4 across various cancers.^100^ This improvement happened through T-cell-specific adenosine 2A receptor A_2A_R signaling (Figure 1).^100^ Besides, increased *Bifidobacterium* abundance, mediated by dietary interventions, augments anti-PD-1 efficacy in mice.^145^ Given these observations, melanoma patients undergoing anti-PD-1 therapy may greatly benefit from the intake of inulin, a type of fructan found in plants that provides a favorable habitat for beneficial bacteria, such as *Bifidobacterium*.^146^

### Conclusion

It is widely recognized that host microbiota has mutual interactions with both innate and adaptive immune cells. These interactions influence the function of innate immune cells and the anti-tumor potential of adaptive immune cells.^147^ Consequently, microbiota-host immune system crosstalk transforms the immune reaction within the tumor microenvironment and affects the efficacy of ICI treatments. Microbiome-based interventions are gradually entering the oncology space, with FMT studies leading the way with the most clinical success.^46^^,^^52^^,^^53^ These studies also highlight the potential for microbiome interventions as an adjunct therapy for mainstream oncology treatments such as ICIs. However, the main mechanisms of action of these treatments remain elusive. Understanding the factors affecting the successful engraftment of the new biome in the new host post-FMT is also critical. Our recent trial in melanoma patients receiving FMT plus anti-PD-1 therapy showed that host features such as body mass index and alpha-diversity can impact engraftment and treatment success in FMT recipients.^46^ These same features could impact engraftment with limited consortia treatment; therefore, more prospective studies are required to determine the external and internal factors that affect engraftment success, such as diet, biological sex, and native microbiota depletion strategies with or without antibiotics before treatment.

**Figure 3:**
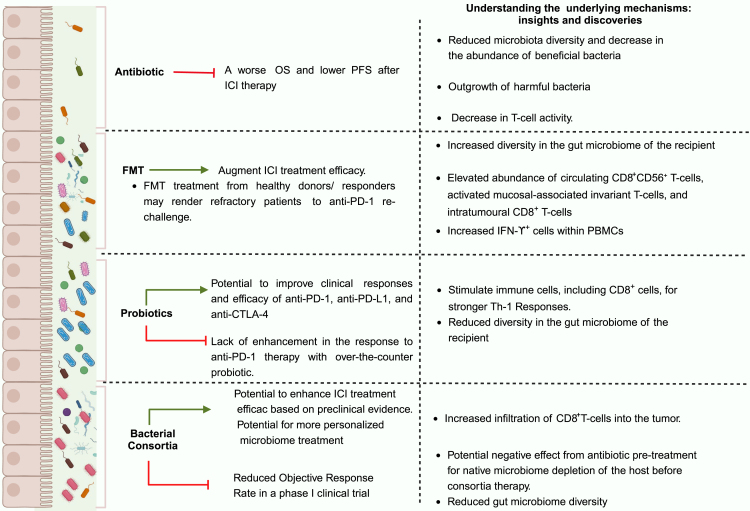
Strategies for modifying gut microbiota and their impacts on immune responses and ICI efficacy While the use of antibiotics and probiotics leads to varying effects on ICI efficacy, the administration of either FMT or consortia demonstrates beneficial impacts on the effectiveness of ICI therapies. The mechanisms underlying these strategies for enhancing and reducing ICI treatment efficacy involve the eradication of harmful bacteria, activation of immune cells, and suppression of activated T cells. ICIs, immune checkpoint inhibitors; OS, overall survival; PFS, progression-free survival; FMT, fecal microbiota transplantation; PBMCs, peripheral blood mononuclear cells; Th-1, T helper-1.

This review presented various preclinical and clinical research highlighting essential bacteria and the mechanisms originating from the microbiota that influence anti-tumor responses and the effectiveness of ICI treatments. Additionally, we reviewed the role of elevated levels of *Bacillota*/*Bacteroides* ratio, *A. muciniphila*, and probiotics in shaping the outcomes of ICI therapies and their potential implications for future research and therapeutic strategies. Treatment approaches that integrate gut microbiota with ICI, including antibiotic therapy, consumption of probiotics, FMT, and bacterial consortia, could pave the way for gut microbiota and their metabolites to emerge as potent adjuncts for ICI therapies (Figure 3).

The next frontier in microbiome research is to design optimal immune-stimulatory consortia that can activate anti-tumor immunity without increasing toxicity toward normal tissues. Certain bacterial families such as Lachnospiraceae,^56^ Ruminococcaceae,^56^^,^^57^ Oscillospiraceae,^50^^,^^55^^,^^58^^,^^59^ and Akkermansiaceae^43^ have immune-stimulatory features and are often enriched in responder cancer patients after ICI treatment. However, it is critical to design future microbiome-based therapies around specific functions rather than the presence or absence of particular species to increase the success of ICI treatment. This theory is supported by the early successes of FMT in the clinic over limited consortia products.

The main limitation of microbiome studies in oncology arises from the focus on bacterial composition over function and the expected lack of consistency in detecting critical bacteria associated with tumor response. Various factors can be responsible for this inconsistency, including differences in geography and population, lifestyle habits, and limited patient sample sizes. More importantly, variations in sample collection, sequencing technologies, and data analysis approaches can greatly influence the microbiome data. A recent publication that re-analyzed the data from a large-scale microbiome study of 33 different cancer types found fundamental errors in the analysis,^148^ demonstrating the lack of established analytical tools in microbiome analysis. Given the inconsistencies of the field, any signature prediction in the context of oncology studies should be tested experimentally and verified in prospective clinical trials. Therefore, it is essential to establish standardized protocols to compare and integrate findings from distinct studies.^64^^,^^149^ Finally, it is crucial to recognize that the functional microbial pathways and microbiome-generated metabolites such as SCFAs may be the common feature among responder patients in different geographic areas exposed to different environmental factors such as unique diets. Thus, more prospective research focusing on these critical factors is required to develop the next generation of microbiome-based interventions for oncology patients.
